# Convergence analysis on a modified generalized alternating direction method of multipliers

**DOI:** 10.1186/s13660-018-1721-z

**Published:** 2018-06-08

**Authors:** Sha Lu, Zengxin Wei

**Affiliations:** 1 School of Science, East China University of Science and Technology, Shanghai, China; 2 School of Mathematics and Statistics, Guangxi Teachers Education University, Nanning, China; 3 School of Mathematics and Information Science, Guangxi University, Nanning, China

**Keywords:** Convex optimization, Augmented Lagrangian function, Alternating direction method of multipliers, Semi-proximal terms

## Abstract

The alternating direction method of multipliers (ADMM) is one of the most powerful and successful methods for solving convex composite minimization problem. The generalized ADMM relaxes both the variables and the multipliers with a common relaxation factor in $(0,2)$, which has the potential of enhancing the performance of the classic ADMM. Very recently, two different variants of semi-proximal generalized ADMM have been proposed. They allow the weighting matrix in the proximal terms to be positive semidefinite, which makes the subproblems relatively easy to evaluate. One of the variants of semi-proximal generalized ADMMs has been analyzed theoretically, but the convergence result of the other is not known so far. This paper aims to remedy this deficiency and establish its convergence result under some mild conditions in the sense that the relaxation factor is also restricted into $(0,2)$.

## Introduction

Let $\mathcal{X}$, $\mathcal{Y}$, and $\mathcal{Z}$ be real finite dimensional Euclidean spaces with the inner product $\langle\cdot, \cdot\rangle$ and its induced norm $\Vert\cdot\Vert$. In this paper, we consider the following convex composite problem with coupled linear equality constraint:

1 $$ \begin{aligned} &\min_{x\in\mathcal{X},y\in\mathcal{Y}} \quad f(x)+g(y) \\ &\quad\text{s.t.}\quad Ax+By=c, \end{aligned} $$

where $f: \mathcal{X}\rightarrow(-\infty,+\infty]$ and $g: \mathcal {Y}\rightarrow(-\infty,+\infty]$ are closed proper convex functions, $A: \mathcal{X} \rightarrow\mathcal{Z}$ and $B: \mathcal{Y} \rightarrow\mathcal{Z}$ are linear operators, and $c\in\mathcal{Z}$ is given. Many applications arising in various areas may have mathematical models with the form of (1), such as image processing, compressed sensing, and statistical learning. Denote $A^{*}$ and $B^{*}$ as the adjoint of *A* and *B*, respectively. Then the dual of problem (1) takes the form

2 $$ \max_{\lambda\in\mathcal{Z}}\bigl\{ -f^{*}\bigl(A^{*}\lambda\bigr)-g^{*} \bigl(B^{*}\lambda \bigr)+\langle\lambda,c\rangle\bigr\} , $$

where $f^{*}(\cdot)$ (resp. $g^{*}(\cdot)$) is a Fenchel conjugate function of *f* (resp. *g*). Under Slater’s constraint qualification, it is known that $(\bar{x},\bar{y})$ is a solution to problem (1) if and only if there exists a Lagrangian multiplier *λ̄* such that the triple $(\bar{x},\bar{y};\bar{\lambda})$ is a solution to the following Karush–Kuhn–Tucker (KKT) conditions system:

3 $$ \textstyle\begin{cases} 0\in\partial f(x)-A^{*}\lambda, \\ 0\in\partial g(y)-B^{*}\lambda,\\ Ax+By-c=0. \end{cases} $$

The augmented Lagrangian function associated with (1) is defined as

$$\mathcal{L}_{\sigma}(x,y;\lambda):=f(x)+g(y)-\langle\lambda, Ax+By-c \rangle +\frac{\sigma}{2} \Vert Ax+By-c \Vert ^{2}, $$

where $\lambda\in\mathcal{Z}$ is a multiplier and $\sigma>0$ is a penalty parameter. Given $(x_{k},y_{k})$, the classic augmented Lagrangian algorithm takes the form to derive the next pair $(x_{k+1},y_{k+1})$: 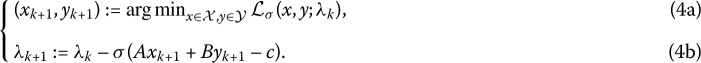 For solving subproblem (4a), we must minimize the function with strongly coupled quadratic term, which makes it hard to solve especially in large-scale problems. By noticing the individual structure of *f* and *g* in problem (1), one effective approach is the alternating direction method of multipliers (abbreviated as ADMM) that for $k=0,1,\ldots$ , 
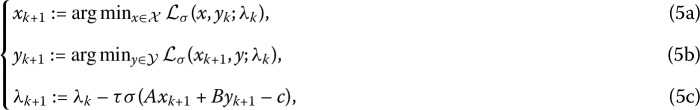
 where *τ* is a step-length which can be chosen in $(0,(1+\sqrt {5})/2)$. The advantage of alternating technique lies in decomposing a large problem into several smaller pieces via its favorable structure, and then solving them accordingly.

The classic ADMM algorithm was originated by Glowinski and Marroco [1], Gabay and Mercier [2] in the mid-1970s. Gabay [3] showed that the classic ADMM with the $\tau=1$ is a special case of the Douglas–Rachford splitting method for monotone operators in the early 1980s. Later, in [4], Eckstein and Bertsekas showed that the Douglas–Rachford splitting method is actually a special case of the proximal point algorithm. The variant of proximal ADMM was proposed by Eckstein [5], which ensures that each subproblem enjoys a unique solution by introducing an additional proximal term. This technique improves the behavior of the objective functions in the iteration subproblems and thus ameliorates the convergent property of the whole algorithm. He et al. [6] in turn showed that the proximal term can be chosen differently pre-iteration. Furthermore, Fazel et al. [7] gave a deep investigation and proved that the proximal term can be chosen to be positive semidefinite, which allows more flexible applications. One may refer to [8] for a note on the historical development of the ADMM, and some further research on ADMM can be seen in [9, 10], etc.

Another contribution of Eckstein and Bertsekas [4] is the designing of a generalized ADMM based on a generalized proximal point algorithm. Very recently, combining the idea of semi-proximal terms, Xiao et al. [11] proposed a semi-proximal generalized ADMM for convex composite conic programming, and numerically illustrated that their proposed method is very promising for solving doubly nonnegative semi-positive definite programming. The method of Xiao et al. [11] relaxed all the variables with a factor of $(0,2)$, which has the potential of enhancing the performance of the classic ADMM. Additionally, in [11], Xiao et al. also developed another variant of semi-proximal generalized ADMM with different semi-proximal terms, but its convergence property has not been investigated so far. This paper targets to prove the global convergence of this semi-proximal generalized ADMM under some mild conditions, which may bring some theoretical foundations in some potential practical applications.

The rest of this paper is organized as follows. In Sect. 2, we present some preliminary results and review some variants of ADMMs. In Sect. 3, we establish the global convergence of the generalized semi-proximal ADMM with semi-proximal terms. In Sect. 4, we conclude this paper with some remarks.

## Preliminaries

In this section, we provide some basic concepts and give a quick review of some variants of generalized ADMMs which will be used in the subsequent developments.

### Basic concepts

Let $\mathcal{E}$ be a finite dimensional real Euclidean space with the inner product and the associated norm denoted by $\langle\cdot ,\cdot\rangle$ and $\Vert\cdot\Vert$, respectively. Let $f:{\mathcal {E}}\rightarrow(-\infty,+\infty]$ be a closed proper convex function. The effective domain of *f* is defined as $\operatorname{dom} f= \{x\in {\mathcal {E}}| f(x)<+\infty\}$. The subdifferential of *f* is the operator defined as $\partial f(x)=\{ x^{*}|f(z)\geq f(x)+\langle x^{*},z-x\rangle, \forall z\in {\mathcal {E}}\}$, and it is simply denoted by $\partial f(x)$. Obviously, $\partial f(x)$ is a closed convex set while it is not empty. The point-to-set operator $\partial f:x\rightarrow\partial f(x)$ is trivially monotone, i.e., for any $x,y\in {\mathcal {E}}$ such that $\partial f(x)$ and $\partial f(y)$ are not empty, it holds that $\langle x-y, u-v\rangle\geq \Vert x-y \Vert ^{2}_{\Sigma}$ for all $u\in\partial f(x)$ and $v\in\partial f(y)$, where $\Sigma:\mathcal{E}\rightarrow\mathcal{E}$ is a self-adjoint positive semidefinite linear operator. The Fenchel conjugate of a function *f* at $y\in {\mathcal {E}}$ is defined as

$$f^{*}(y):=\sup_{x}\bigl\{ \langle x,y\rangle-f(x)\bigr\} =-\inf _{x}\bigl\{ f(x)-\langle x,y\rangle\bigr\} . $$

It is well known in [12] that the conjugate function $f^{*}(y)$ is always convex and closed, proper if and only if *f* is proper. Furthermore, $(\operatorname{cl} f)^{*}=f^{*}$ and $f^{**}= \operatorname{cl} f$, where cl*f* denotes the closed function of *f*, i.e., the epigraph of cl*f* is a closure of the epigraph of the convex function *f*.

Assuming that the KKT system (3) is not empty, then the dual problem (2) can be solved by using the splitting method to solve the following inclusion problem:

6 $$ 0\in(T_{1}+T_{2}) (\lambda) $$

with

7 $$ T_{1}(\lambda)=c- A\partial f^{*}\bigl(-A^{*}\lambda\bigr) \quad\text{and}\quad T_{2}(\lambda)=-B\partial g^{*}\bigl(-B^{*}\lambda \bigr). $$

It is easy to see that both $T_{1}$ and $T_{2}$ are maximal monotone operators. To solve (6), an equivalent form of the generalized proximal point algorithm of Eckstein and Bertsekas [4] with any initial point $v_{0}$ is that

8 $$ \textstyle\begin{cases} v_{k+1}=(1-\rho)v_{k}+\rho[J_{\sigma T_{1}}(2J_{\sigma T_{2}}-I)v_{k}+(I-J_{\sigma T_{2}})v_{k} ],\\ \lambda_{k+1}=J_{\sigma T_{2}}(v_{k+1}), \end{cases} $$

where $\rho\in(0,2)$ and $J_{\sigma T}=( I+\sigma T)^{-1}$ is the so-called resolvent operator.

### Generalized ADMM

Eckstein and Bertsekas [4] further showed that the iterative framework (8) is equivalent to the following iterative scheme while it is used to solve the minimization problem (1): 
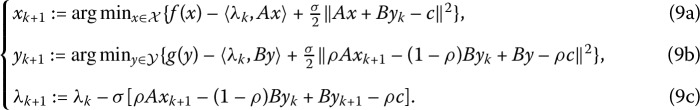
 Obviously, the classic ADMM (5a)–(5c) with $\tau =1$ is exactly the generalized ADMM with $\rho=1$.

Furthermore, Chen [13] showed that the generalized ADMM (9a)–(9c) is equivalent to the following ADMM scheme with initial point $\tilde{w}_{0}=(\tilde{x}_{0},\tilde{y}_{0};\tilde{\lambda}_{0})$, and the parameter $\rho\in(0,2)$ is transformed into a relaxation factor: 
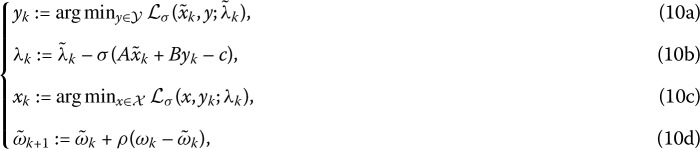
 where $\omega_{k}=(x_{k},y_{k};\lambda_{k})$ and $\tilde{\omega}_{k}=(\tilde{x}_{k},\tilde{y}_{k};\tilde{\lambda}_{k})$. More details on the equivalence of these two methods are also given in the Ph.D. thesis [13].

### Proximal ADMM

In order to broaden the capability of the classic ADMM, Eckstein [5] added a proximal term to each subproblem, which reduced to 
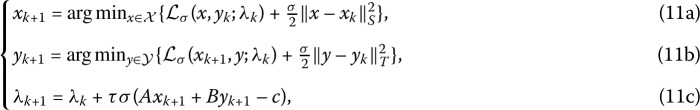
 where *S* and *T* are positive definite matrices. Moreover, Fazel et al. [7] further illustrated that both weighting matrices can be chosen as positive semidefinite so that it can be applied in more practical situations. For more details on its convergence results, one can refer to [7] and the references therein.

It can be observed that the subproblems in the generalized ADMM schemes (10a)–(10d) may not admit solutions because *A* or *B* is not assumed to be row full-rank. One natural way to fix this problem is to add proximal terms to these subproblems. Very recently, Xiao et al. [11] suggested a couple of approaches to achieve this purpose. One of them is to add the semi-proximal terms $\frac{1}{2} \Vert x-x_{k-1} \Vert_{S}^{2}$ and $\frac{1}{2} \Vert y-y_{k-1} \Vert_{T}^{2}$ to the subproblems for computing $x_{k}$ and $y_{k}$, i.e., 
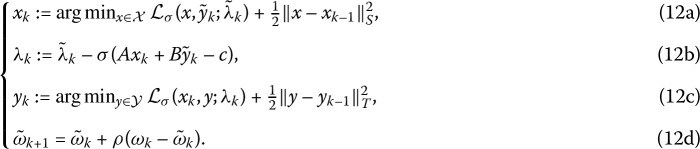
 Another one is to add the proximal terms $\frac{1}{2} \Vert x-\tilde{x}_{k} \Vert_{{\mathcal {S}}}$ and $\frac{1}{2} \Vert y-\tilde{y}_{k} \Vert _{T}^{2}$, i.e., 
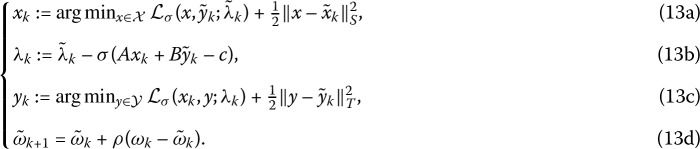
 Compared with the traditional proximal approach (12a)–(12d), the semi-proximal terms in (13a)–(13d) are more natural in the sense that the most recently updated values of variables are involved. Actually, the global convergence of the iterative framework (13a)–(13d) has been analyzed in [11], and the corresponding numerical results illustrated that the proposed method can solve these problems not only effectively but also efficiently. In this paper, we particularly concentrate on the convergence analysis of the corresponding algorithm based on the former iterative framework to solve the separable convex minimization problem (1).

## Global convergence

This section is devoted to analyzing the global convergence of the generalized ADMM based on the iterative framework (12a)–(12d) Since *f* and *g* are both closed proper convex functions, it is known that *∂f* and *∂g* are maximal monotone mappings [14], and then there exist a couple of self-adjoint positive semidefinite linear operators $\Sigma_{f}:{\mathcal {X}}\to {\mathcal {X}}$ and $\Sigma_{g}:{\mathcal {Y}}\to {\mathcal {Y}}$ such that, for any $x,x'\in {\mathcal {X}}$ and $y,y'\in {\mathcal {Y}}$ with $u\in\partial f(x)$, $u'\in \partial f(x')$, $v\in\partial g(y)$, and $v'\in\partial g(y')$,

14 $$ \bigl\langle u-u',x-x'\bigr\rangle \geq \bigl\Vert x-x' \bigr\Vert _{\Sigma_{f}}^{2} \quad \mbox{and} \quad \bigl\langle v-v',y-y'\bigr\rangle \geq \bigl\Vert y-y' \bigr\Vert _{\Sigma_{g}}^{2}. $$

First, we state the detailed steps of the generalized ADMM with semi-proximal terms (abbreviate it as sPGADM) as follows.

### Algorithm sPGADM

Set $\rho\in(0,2)$, $\sigma>0$. Choose $S:{\mathcal {X}}\to {\mathcal {X}}, T:{\mathcal {Y}}\to {\mathcal {Y}}$ such that $\sum_{f}+S+A^{*}A\succ0$ and $\sum_{g}+T+B^{*}B\succ0$. Input an initial point $\tilde{\omega}_{0}=(\tilde{x}_{0},\tilde{y}_{0};\tilde {\lambda}_{0})\in {\mathcal {X}}\times {\mathcal {Y}}\times {\mathcal {Z}}$. For $k=1,2,\ldots$ ,

*Step 1 (main step).* Compute 
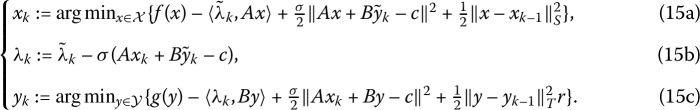


*Step 2 (relaxation step).* Compute

16 $$ \tilde{\omega}_{k+1}=\tilde{\omega}_{k}+\rho( \omega_{k}-\tilde {\omega}_{k}). $$

Before deducing the convergence property of sPGADM, we do some preparations to facilitate the later analysis. Firstly, we make the following assumption.

### Assumption A

There exists at least one vector $(\bar{x},\bar{y};\bar{\lambda })\in {\mathcal {X}}\times {\mathcal {Y}}\times {\mathcal {Z}}$ such that the KKT system (3) is satisfied.

We now let $\{(x_{k},y_{k};\lambda_{k})\}$ be the sequence generated by sPGADM and $(\bar{x},\bar{y};\bar{\lambda})$ be a solution of the KKT system (3). For a more convenient discussion, we denote $x_{k}^{e}=x_{k}-\bar{x}$, $y_{k}^{e}=y_{k}-\bar{y}$, and $\lambda_{k}^{e}=\lambda_{k}-\bar{\lambda}$.

The first-order optimality condition of (15a) can be expressed as

$$A^{*}\tilde{\lambda}_{k}-\sigma A^{*}(Ax_{k}+B\tilde {y}_{k}-c)-S(x_{k}-x_{k-1})\in\partial f(x_{k}), $$

which combined with (15b) yields

17 $$\begin{aligned} A^{*}\lambda_{k}-S(x_{k}-x_{k-1})\in \partial f(x_{k}) \end{aligned}$$

and

18 $$\begin{aligned} A^{*}\lambda_{k+1}-S(x_{k+1}-x_{k})\in \partial f(x_{k+1}). \end{aligned}$$

Since *x̄* and *λ̄* satisfy the KKT system (3), then we obtain from (14) that

$$\bigl\langle A^{*}\lambda_{k+1}-S(x_{k+1}-x_{k})-A^{*} \bar{\lambda}, x_{k+1}-\bar{x}\bigr\rangle \geq \Vert x_{k+1}- \bar{x} \Vert ^{2}_{\Sigma_{f}}, $$

or, equivalently, by noting the definition of $x^{e}_{k}$,

19 $$\begin{aligned} \bigl\langle \lambda_{k+1}^{e}, Ax_{k+1}^{e}\bigr\rangle - \bigl\langle S(x_{k+1}-x_{k}),x_{k+1}^{e} \bigr\rangle \geq \bigl\Vert x_{k+1}^{e} \bigr\Vert ^{2}_{\Sigma_{f}}. \end{aligned}$$

Similarly, the first-order optimality condition of (15c) can also be described as

20 $$\begin{aligned} B^{*}\lambda_{k}-\sigma B^{*}(Ax_{k}+By_{k}-c)-T(y_{k}-y_{k-1}) \in\partial g(y_{k}). \end{aligned}$$

Thus, from the monotone property (14), we get

$$\bigl\langle B^{*}\lambda_{k}-\sigma B^{*}(Ax_{k}+By_{k}-c)-T(y_{k}-y_{k-1})-B^{*} \bar {\lambda}, y_{k}-\bar{y}\bigr\rangle \geq \Vert y_{k}- \bar{y} \Vert ^{2}_{\Sigma_{g}}, $$

or, equivalently,

21 $$\begin{aligned} \bigl\langle \lambda_{k}^{e}, By_{k}^{e}\bigr\rangle - \bigl\langle \sigma(Ax_{k}+By_{k}-c), By_{k}^{e}\bigr\rangle -\bigl\langle T(y_{k}-y_{k-1}),y_{k}^{e} \bigr\rangle \geq \bigl\Vert y_{k}^{e} \bigr\Vert ^{2}_{\Sigma_{g}}. \end{aligned}$$

Adding two sides of (19) and (21) implies

$$\begin{aligned} & \bigl\langle \lambda_{k+1}^{e}, Ax_{k+1}^{e}\bigr\rangle +\bigl\langle \lambda_{k}^{e}, By_{k}^{e}\bigr\rangle -\bigl\langle \sigma(Ax_{k}+By_{k}-c), By_{k}^{e}\bigr\rangle \\ & \qquad{}-\bigl\langle S(x_{k+1}-x_{k}),x_{k+1}^{e} \bigr\rangle -\bigl\langle T(y_{k}-y_{k-1}),y_{k}^{e} \bigr\rangle \\ &\quad\geq \bigl\Vert x_{k+1}^{e} \bigr\Vert ^{2}_{\Sigma_{f}}+ \bigl\Vert y_{k}^{e} \bigr\Vert ^{2}_{\Sigma_{g}}, \end{aligned} $$

which can be equivalently rewritten as

22 $$ \begin{aligned}[b] & \bigl\langle \lambda_{k+1}^{e}, Ax_{k+1}^{e}+By_{k}^{e}\bigr\rangle +\bigl\langle \lambda _{k}^{e} -\lambda_{k+1}^{e}, By_{k}^{e}\bigr\rangle -\bigl\langle \sigma (Ax_{k}+By_{k}-c), By_{k}^{e}\bigr\rangle \\ & \qquad{}-\bigl\langle S(x_{k+1}-x_{k}),x_{k+1}^{e} \bigr\rangle -\bigl\langle T(y_{k}-y_{k-1}),y_{k}^{e} \bigr\rangle \\ &\quad\geq \bigl\Vert x_{k+1}^{e} \bigr\Vert ^{2}_{\Sigma_{f}}+ \bigl\Vert y_{k}^{e} \bigr\Vert ^{2}_{\Sigma_{g}}. \end{aligned} $$

Note that the first term on the left-hand side of (22) can be reorganized as

23 $$ \begin{aligned}[b] \bigl\langle \lambda_{k+1}^{e}, Ax_{k+1}^{e}+By_{k}^{e}\bigr\rangle &= \bigl\langle \lambda _{k+1}^{e}+\sigma(1-\rho)Ax_{k+1}^{e}, Ax_{k+1}^{e}+By_{k}^{e}\bigr\rangle \\ & \quad{}-\sigma(1-\rho)\bigl\langle Ax_{k+1}^{e}+By_{k}^{e}, Ax_{k+1}^{e}+By_{k}^{e}\bigr\rangle \\ &\quad{}+\sigma(1-\rho)\bigl\langle By_{k}^{e}, Ax_{k+1}^{e}+By_{k}^{e}\bigr\rangle . \end{aligned} $$

Then, together with $\lambda_{k}^{e}-\lambda_{k+1}^{e}=\lambda_{k}-\lambda_{k+1}$, (22) is transformed equally into

24 $$ \begin{aligned}[b] &\bigl\langle \lambda_{k+1}^{e}+ \sigma(1-\rho)Ax_{k+1}^{e}, Ax_{k+1}^{e}+By_{k}^{e} \bigr\rangle +\bigl[\sigma(1-\rho)\bigl\langle By_{k}^{e}, Ax_{k+1}^{e}+By_{k}^{e}\bigr\rangle \\ &\qquad{}+\bigl\langle \lambda_{k}^{e}-\lambda_{k+1}^{e}, By_{k}^{e}\bigr\rangle -\bigl\langle \sigma(Ax_{k}+By_{k}-c), By_{k}^{e}\bigr\rangle \bigr] \\ &\qquad{}-\bigl\langle S(x_{k+1}-x_{k}),x_{k+1}^{e} \bigr\rangle -\bigl\langle T(y_{k}-y_{k-1}),y_{k}^{e} \bigr\rangle \\ &\quad\geq \bigl\Vert x_{k+1}^{e} \bigr\Vert ^{2}_{\Sigma_{f}}+ \bigl\Vert y_{k}^{e} \bigr\Vert ^{2}_{\Sigma_{g}}+\sigma(1-\rho) \bigl\Vert Ax_{k+1}^{e}+By_{k}^{e} \bigr\Vert ^{2}. \end{aligned} $$

The following two lemmas play a fundamental role in our convergence analysis.

### Lemma 3.1

*Let* $\{(x_{k}, y_{k}; \lambda_{k})\}$ *be the sequence generated from algorithm sPGADM*, *and* $\{\bar{x},\bar{y};\bar{\lambda}\}$ *is a solution of the KKT system*. *For* $k=1,2,\ldots$ , *it holds that*

25 $$ \begin{aligned}[b] &\bigl\langle \lambda_{k+1}^{e}+ \sigma(1-\rho)Ax_{k+1}^{e}, Ax_{k+1}^{e}+By_{k}^{e} \bigr\rangle \\ &\quad=-\frac{\sigma\rho}{2} \bigl\Vert Ax_{k+1}^{e}+By_{k}^{e} \bigr\Vert ^{2}-\frac{1}{2\sigma\rho} \bigl[ \bigl\Vert \lambda_{k+1}^{e}+\sigma (1-\rho)Ax_{k+1}^{e} \bigr\Vert ^{2} \\ &\qquad{}- \bigl\Vert \lambda_{k}^{e}-\sigma(1- \rho)Ax_{k}^{e} \bigr\Vert ^{2}\bigr]. \end{aligned} $$

### Proof

From (15b) and (16), we have

$$\tilde{\lambda}_{k+1}=\lambda_{k}+(\rho-1) ( \lambda_{k}-\tilde{\lambda}_{k}) =\lambda_{k}- \sigma(\rho-1) (Ax_{k}+B\tilde{y}_{k}-c) $$

and

26 $$ \begin{aligned}[b] \lambda_{k+1}&=\tilde{ \lambda}_{k+1}-\sigma(Ax_{k+1}+B\tilde {y}_{k+1}-c) \\ &=\lambda_{k}-\sigma(\rho-1) (Ax_{k}+B \tilde{y}_{k}-c)-\sigma \bigl[Ax_{k+1} +B\bigl( \tilde{y}_{k}+\rho(y_{k}-\tilde{y}_{k})\bigr)-c \bigr] \\ &=\lambda_{k}-\sigma\rho(Ax_{k+1}+By_{k}-c)-\sigma( \rho-1) (Ax_{k}-Ax_{k+1}). \end{aligned} $$

Thus,

$$\begin{aligned}&\bigl(\lambda_{k+1}^{e}+ \sigma(1-\rho)Ax_{k+1}^{e}\bigr)-\bigl(\lambda_{k}^{e}+ \sigma (1-\rho) Ax_{k}^{e}\bigr) \\ &\quad=(\lambda_{k+1}-\lambda_{k})+\sigma(1-\rho ) (Ax_{k+1}-Ax_{k}) \\ &\quad=-\sigma\rho(Ax_{k+1}+By_{k}-c)-\sigma(\rho-1) (Ax_{k}-Ax_{k+1}) +\sigma(1-\rho) (Ax_{k+1}-Ax_{k}) \\ &\quad=-\sigma\rho(Ax_{k+1}+By_{k}-c). \end{aligned} $$

By using (26), we get

$$\bigl(\lambda_{k+1}^{e}+\sigma(1-\rho)Ax_{k+1}^{e} \bigr)+\sigma\rho(Ax_{k+1}+By_{k}-c)= \lambda_{k}^{e}+ \sigma(1-\rho)Ax_{k}^{e}. $$

Together with the basic relation

27 $$\begin{aligned} 2\langle u,v \rangle= \Vert u \Vert ^{2}+ \Vert v \Vert ^{2}- \Vert u-v \Vert ^{2}= \Vert u+v \Vert ^{2}- \Vert u \Vert ^{2}- \Vert v \Vert ^{2}, \end{aligned}$$

it implies that

$$\begin{aligned} &2\bigl\langle \lambda_{k+1}^{e}+ \sigma(1-\rho)Ax_{k+1}^{e}, \sigma\rho (Ax_{k+1}+By_{k}-c) \bigr\rangle \\ &\quad= \bigl\Vert \lambda_{k}^{e}+\sigma(1- \rho)Ax_{k}^{e} \bigr\Vert ^{2}- \bigl\Vert \lambda_{k+1}^{e}+\sigma(1-\rho)Ax_{k+1}^{e} \bigr\Vert ^{2} \\ & \qquad{}-\sigma^{2}\rho^{2} \Vert Ax_{k+1}+By_{k}-c \Vert ^{2}, \end{aligned} $$

which is equivalent to

28 $$ \begin{aligned}[b] & \bigl\langle \lambda_{k+1}^{e}+ \sigma(1-\rho)Ax_{k+1}^{e}, Ax_{k+1}+By_{k}-c \bigr\rangle \\ &\quad=\frac{1}{2\sigma\rho} \bigl\Vert \lambda_{k}^{e}+ \sigma (1-\rho)Ax_{k}^{e} \bigr\Vert ^{2} - \frac{1}{2\sigma\rho} \bigl\Vert \lambda_{k+1}^{e}+\sigma(1- \rho)Ax_{k+1}^{e} \bigr\Vert ^{2} \\ & \qquad{}-\frac{\sigma\rho}{2} \Vert Ax_{k+1}+By_{k}-c \Vert ^{2}. \end{aligned} $$

Since $\{\bar{x},\bar{y};\bar{\lambda}\}$ satisfies the KKT system (3) that

$$A\bar{x}+B\bar{y}=c,\qquad Ax_{k+1}+By_{k}-c=Ax^{e}_{k+1}+By^{e}_{k}, $$

then from (28) we get (25). □

### Lemma 3.2

*Let* $\{(x_{k}, y_{k}; \lambda_{k})\}$ *be a sequence generated from algorithm sPGADM*, *and* $\{\bar{x},\bar{y};\bar{\lambda}\}$ *be a solution of the KKT system*. *For* $k=1,2,\ldots $ , *it holds that*

29 $$ \begin{aligned}[b] & \sigma(1-\rho)\bigl\langle By_{k}^{e}, Ax_{k+1}^{e}+By_{k}^{e} \bigr\rangle +\bigl\langle \lambda_{k}^{e} - \lambda_{k+1}^{e}, By_{k}^{e}\bigr\rangle - \bigl\langle \sigma (Ax_{k}+By_{k}-c), By_{k}^{e} \bigr\rangle \\ &\quad\leq-\frac{2-\rho}{\rho} \Vert x_{k+1}-x_{k} \Vert ^{2}_{\Sigma_{f}}-\frac{2-\rho}{2\rho} \Vert x_{k+1}-x_{k} \Vert ^{2}_{S}+\frac{2-\rho}{2\rho} \Vert x_{k}-x_{k-1} \Vert ^{2}_{S} \\ & \qquad{}-\frac{\sigma(2-\rho)^{2}}{2\rho} \bigl\Vert Ax_{k+1}^{e}-Ax_{k}^{e} \bigr\Vert ^{2}+ \frac{\sigma(2-\rho)}{2} \bigl\Vert Ax_{k}^{e} \bigr\Vert ^{2}-\frac{\sigma(2-\rho)}{2} \bigl\Vert Ax_{k+1}^{e} \bigr\Vert ^{2}. \end{aligned} $$

### Proof

By using the elementary relation (27), we have

30 $$ \begin{aligned}[b] &\bigl\langle S(x_{k+1}-x_{k})-S(x_{k}-x_{k-1}),x_{k+1}-x_{k} \bigr\rangle \\ &\quad= \Vert x_{k+1}-x_{k} \Vert ^{2}_{S}- \bigl\langle S(x_{k}-x_{k-1}),x_{k+1}-x_{k} \bigr\rangle \\ &\quad\geq \Vert x_{k+1}-x_{k} \Vert ^{2}_{S}- \frac{1}{2}\bigl( \Vert x_{k+1}-x_{k} \Vert ^{2}_{S}+ \Vert x_{k}-x_{k-1} \Vert ^{2}_{S}\bigr) \\ &\quad=\frac{1}{2} \Vert x_{k+1}-x_{k} \Vert ^{2}_{S}-\frac {1}{2} \Vert x_{k}-x_{k-1} \Vert ^{2}_{S}. \end{aligned} $$

Similar to (19), from (14), (17), and (18), we get

$$\bigl\langle A^{*}(\lambda_{k+1}-\lambda_{k})-S(x_{k+1}-x_{k}) +S(x_{k}-x_{k-1}), x_{k+1}-x_{k}\bigr\rangle \geq \Vert x_{k+1}-x_{k} \Vert ^{2}_{\Sigma_{f}}. $$

Then

$$\bigl\langle \lambda_{k+1}-\lambda_{k}, A(x_{k+1}-x_{k}) \bigr\rangle - \bigl\langle S(x_{k+1}-x_{k})-S(x_{k}-x_{k-1}),x_{k+1}-x_{k} \bigr\rangle \geq \Vert x_{k+1}-x_{k} \Vert ^{2}_{\Sigma_{f}}, $$

which together with (30) implies

31 $$ \begin{aligned}[b] & \bigl\langle \lambda_{k+1}- \lambda_{k}, A(x_{k+1}-x_{k})\bigr\rangle \\ &\quad\geq \frac{1}{2} \Vert x_{k+1}-x_{k} \Vert ^{2}_{S} -\frac{1}{2} \Vert x_{k}-x_{k-1} \Vert ^{2}_{S}+ \Vert x_{k+1}-x_{k} \Vert ^{2}_{\Sigma_{f}}. \end{aligned} $$

Notice that from (26)

$$\sigma\bigl(Ax_{k+1}^{e}+By_{k}^{e} \bigr)= -\rho^{-1}\bigl[\lambda_{k+1}-\lambda _{k}+ \sigma(1-\rho) (Ax_{k+1}-Ax_{k})\bigr]. $$

Combining it with (31), we obtain

32 $$ \begin{aligned}[b] &\sigma\bigl\langle Ax_{k}^{e}-Ax_{k+1}^{e},Ax_{k+1}^{e}+By_{k}^{e} \bigr\rangle \\ &\quad=-\rho^{-1}\bigl\langle Ax_{k}^{e}-Ax_{k+1}^{e}, \lambda_{k+1}-\lambda _{k}+\sigma(1-\rho) (Ax_{k+1}-Ax_{k}) \bigr\rangle \\ &\quad=\rho^{-1}\bigl\langle Ax_{k+1}^{e}-Ax_{k}^{e}, \lambda_{k+1}-\lambda _{k}\bigr\rangle +\rho^{-1} \sigma(1-\rho) \bigl\Vert Ax_{k+1}^{e}-Ax_{k}^{e} \bigr\Vert ^{2} \\ &\quad\geq\frac{1}{\rho} \Vert x_{k+1}-x_{k} \Vert ^{2}_{\Sigma_{f}}+\frac{1}{2\rho} \Vert x_{k+1}-x_{k} \Vert ^{2}_{S} -\frac{1}{2\rho} \Vert x_{k}-x_{k-1} \Vert ^{2}_{S} \\ & \qquad{}+\frac{\sigma(1-\rho)}{\rho} \bigl\Vert Ax_{k+1}^{e}-Ax_{k}^{e} \bigr\Vert ^{2}. \end{aligned} $$

Also from (26)

33 $$ \begin{aligned}[b] & \sigma(1-\rho) \bigl(Ax_{k+1}^{e}+By_{k}^{e}\bigr)+\bigl( \lambda_{k}^{e}-\lambda _{k+1}^{e}\bigr)- \sigma(Ax_{k}+By_{k}-c) \\ &\quad=\sigma(1-\rho) \bigl(Ax_{k+1}^{e}+By_{k}^{e} \bigr)+\bigl[\sigma\rho (Ax_{k+1}+By_{k}-c)+\sigma(\rho-1) (Ax_{k}-Ax_{k+1})\bigr] \\ &\qquad{} -\sigma(Ax_{k}+By_{k}-c) \\ &\quad=\sigma(\rho-2) \bigl(Ax_{k}^{e}-Ax_{k+1}^{e} \bigr). \end{aligned} $$

Then, since $\rho\in(0,2)$, by using (33), (32), and (27) successively, we deduce that

34 $$ \begin{aligned}[b] & \sigma(1-\rho)\bigl\langle Ax_{k+1}^{e}+By_{k}^{e}, By_{k}^{e}\bigr\rangle +\bigl\langle \lambda_{k}^{e}- \lambda_{k+1}^{e}, By_{k}^{e}\bigr\rangle - \sigma\bigl\langle Ax_{k}+By_{k}-c, By_{k}^{e} \bigr\rangle \\ &\quad=\sigma(\rho-2)\bigl\langle Ax_{k}^{e}-Ax_{k+1}^{e}, By_{k}^{e}\bigr\rangle \\ &\quad=-\sigma(2-\rho)\bigl\langle Ax_{k}^{e}-Ax_{k+1}^{e}, Ax_{k+1}^{e}+By_{k}^{e}\bigr\rangle + \sigma(2-\rho)\bigl\langle Ax_{k}^{e}-Ax_{k+1}^{e}, Ax_{k+1}^{e}\bigr\rangle \\ &\quad\leq-(2-\rho)\biggl[\frac{1}{\rho} \Vert x_{k+1}-x_{k} \Vert ^{2}_{\Sigma_{f}}+\frac{1}{2\rho} \Vert x_{k+1}-x_{k} \Vert ^{2}_{S}- \frac{1}{2\rho} \Vert x_{k}-x_{k-1} \Vert ^{2}_{S} \\ & \qquad{}+\frac{\sigma(1-\rho)}{\rho} \bigl\Vert Ax_{k+1}^{e}-Ax_{k}^{e} \bigr\Vert ^{2}\biggr] \\ & \qquad{}+\frac{\sigma(2-\rho)}{2}\bigl[ \bigl\Vert Ax_{k}^{e} \bigr\Vert ^{2} - \bigl\Vert Ax_{k}^{e}-Ax_{k+1}^{e} \bigr\Vert ^{2} - \bigl\Vert Ax_{k+1}^{e} \bigr\Vert ^{2}\bigr] \\ &\quad=-\frac{2-\rho}{\rho} \Vert x_{k+1}-x_{k} \Vert ^{2}_{\Sigma_{f}}-\frac{2-\rho}{2\rho} \Vert x_{k+1}-x_{k} \Vert ^{2}_{S}+\frac{2-\rho}{2\rho} \Vert x_{k}-x_{k-1} \Vert ^{2}_{S} \\ & \qquad{}-\frac{\sigma(2-\rho)^{2}}{2\rho} \bigl\Vert Ax_{k+1}^{e}-Ax_{k}^{e} \bigr\Vert ^{2} +\frac{\sigma(2-\rho)}{2} \bigl\Vert Ax_{k}^{e} \bigr\Vert ^{2}-\frac{\sigma(2-\rho)}{2} \bigl\Vert Ax_{k+1}^{e} \bigr\Vert ^{2}. \end{aligned} $$

Thus, (29) is true and the proof is completed. □

Now, based on Lemmas 3.1 and 3.2, we define $\phi_{k}$ ($k>0$) as

35 $$ \begin{aligned}[b] \phi_{k}&:= \frac{1}{\sigma\rho} \bigl\Vert \lambda_{k}^{e}+\sigma (1- \rho)Ax_{k}^{e} \bigr\Vert ^{2}+ \bigl\Vert x_{k}^{e} \bigr\Vert ^{2}_{S} + \bigl\Vert y_{k-1}^{e} \bigr\Vert ^{2}_{T} \\ & \quad{}+\frac{2-\rho}{\rho} \Vert x_{k}-x_{k-1} \Vert ^{2}_{S}+\sigma(2-\rho) \bigl\Vert Ax_{k}^{e} \bigr\Vert ^{2}. \end{aligned} $$

The following theorem shows that the sequence $\{\phi_{k}\}_{k>0}$ is monotonically decreasing and the algorithm sPGADM is globally convergent.

### Theorem 3.3

*Assume that the solution set of* (1) *is nonempty and that there exists a vector* $(\bar{x},\bar{y};\bar{\lambda})$ *satisfying the KKT system* (3). *Let* $\phi_{k}(k>0)$ *be defined as* (35), *and let the sequence* $\{(x_{k},y_{k};\lambda_{k})\}$ *be generated from algorithm sPGADM*. *Then*, *for* $k=1,2,\ldots$ ,

36 $$ \begin{aligned}[b] & \phi_{k}- \phi_{k+1} \\ &\quad\geq2 \bigl\Vert x_{k+1}^{e} \bigr\Vert ^{2}_{\Sigma_{f}}+2 \bigl\Vert y_{k}^{e} \bigr\Vert ^{2}_{\Sigma_{g}} +\sigma(2-\rho) \bigl\Vert Ax_{k+1}^{e}+ By_{k}^{e} \bigr\Vert ^{2}+ \Vert x_{k+1}-x_{k} \Vert ^{2}_{S} \\ & \qquad{}+ \Vert y_{k}-y_{k-1} \Vert ^{2}_{T}+\frac {2(2-\rho)}{\rho} \Vert x_{k+1}-x_{k} \Vert ^{2}_{\Sigma_{f}} +\frac{\sigma(2-\rho)^{2}}{\rho} \bigl\Vert Ax_{k}^{e}-Ax_{k+1}^{e} \bigr\Vert ^{2}. \end{aligned} $$

*Furthermore*, *the sequence* $\{(x_{k},y_{k};\lambda_{k})\}$ *converges to a KKT solution* $(\bar{x},\bar{y};\bar{\lambda})$ *to problem* (1).

### Proof

From (27), we have

37 $$\begin{aligned}& \bigl\langle S(x_{k+1}-x_{k}), x_{k+1}^{e} \bigr\rangle =\frac{1}{2}\bigl[ \Vert x_{k+1}-x_{k} \Vert ^{2}_{S} + \Vert x_{k+1}-\bar{x} \Vert ^{2}_{S}- \Vert x_{k}-\bar{x} \Vert ^{2}_{S}\bigr], \end{aligned}$$

38 $$\begin{aligned}& \bigl\langle T(y_{k}-y_{k-1}), y_{k}^{e} \bigr\rangle =\frac{1}{2}\bigl[ \Vert y_{k}-y_{k-1} \Vert ^{2}_{T} + \Vert y_{k}-\bar{y} \Vert ^{2}_{T}- \Vert y_{k-1}-\bar{y} \Vert ^{2}_{T}\bigr]. \end{aligned}$$

Substituting (25), (29), (37), and (38) into the four parts of the left-hand side of (24) respectively, we get

39 $$ \begin{aligned}[b] & \biggl[-\frac{\sigma\rho}{2} \bigl\Vert Ax_{k+1}^{e}+By_{k}^{e} \bigr\Vert ^{2}-\frac{1}{2\sigma\rho} \bigl( \bigl\Vert \lambda_{k+1}^{e}+ \sigma (1-\rho)Ax_{k+1}^{e} \bigr\Vert ^{2} \\ & \qquad{}- \bigl\Vert \lambda_{k}^{e}-\sigma(1- \rho)Ax_{k}^{e} \bigr\Vert ^{2}\bigr)\biggr]- \frac{2-\rho}{\rho} \Vert x_{k+1}-x_{k} \Vert ^{2}_{\Sigma_{f}} \\ & \qquad{}-\frac{2-\rho}{2\rho}\bigl( \Vert x_{k+1}-x_{k} \Vert ^{2}_{S}- \Vert x_{k}-x_{k-1} \Vert ^{2}_{S}\bigr)-\frac{\sigma (2-\rho)^{2}}{2\rho} \bigl\Vert Ax_{k+1}^{e}-Ax_{k}^{e} \bigr\Vert ^{2} \\ & \qquad{}+\frac{\sigma(2-\rho)}{2}\bigl( \bigl\Vert Ax_{k}^{e} \bigr\Vert ^{2}- \bigl\Vert Ax_{k+1}^{e} \bigr\Vert ^{2}\bigr)-\frac{1}{2}\bigl( \Vert x_{k+1}-x_{k} \Vert ^{2}_{S}+ \bigl\Vert x_{k+1}^{e} \bigr\Vert ^{2}_{S}- \bigl\Vert x_{k}^{e} \bigr\Vert ^{2}_{S}\bigr) \\ & \qquad{}-\frac{1}{2}\bigl( \Vert y_{k}-y_{k-1} \Vert ^{2}_{T}+ \bigl\Vert y_{k}^{e} \bigr\Vert ^{2}_{T}- \bigl\Vert y_{k-1}^{e} \bigr\Vert ^{2}_{T}\bigr) \\ &\quad\geq\bigl\langle \lambda_{k+1}^{e}+\sigma(1- \rho)Ax_{k+1}^{e}, Ax_{k+1}^{e}+By_{k}^{e} \bigr\rangle +\bigl[\sigma(1-\rho)\bigl\langle By_{k}^{e}, Ax_{k+1}^{e}+By_{k}^{e}\bigr\rangle \\ & \qquad{}+\bigl\langle \lambda_{k}^{e}-\lambda_{k+1}^{e}, By_{k}^{e}\bigr\rangle -\bigl\langle \sigma(Ax_{k}+By_{k}-c), By_{k}^{e}\bigr\rangle \bigr] \\ & \qquad{}-\bigl\langle S(x_{k+1}-x_{k}),x_{k+1}^{e} \bigr\rangle -\bigl\langle T(y_{k}-y_{k-1}),y_{k}^{e} \bigr\rangle \\ &\quad\geq \bigl\Vert x_{k+1}^{e} \bigr\Vert ^{2}_{\Sigma_{f}}+ \bigl\Vert y_{k}^{e} \bigr\Vert ^{2}_{\Sigma_{g}}+\sigma(1-\rho) \bigl\Vert Ax_{k+1}^{e}+ By_{k}^{e} \bigr\Vert ^{2}. \end{aligned} $$

By the definition of $\phi_{k}(k>0)$ and some simple deformations, it is easy to see that (39) implies (36).

Because $\rho\in(0,2)$, then (35) and (36) deduce that the sequence $\{\phi_{k}\}_{k>0}$ is nonnegative and monotonically nonincreasing. Thus, the boundedness of ${\phi_{k}}$ means that each part on the right hand side of (35), such as $\{ \Vert\lambda _{k}^{e}+\sigma(1-\rho)Ax_{k}^{e} \Vert\}$, $\{ \Vert x_{k}^{e} \Vert_{S}\}$, $\{ \Vert y_{k-1}^{e} \Vert_{T}\}$, $\{ \Vert x_{k}-x_{k-1} \Vert_{S}\}$, and $\{ \Vert Ax_{k}^{e} \Vert\}$, is bounded. Also, from (36), it gives that when $k\rightarrow\infty$,

40 $$ \begin{gathered} \bigl\Vert x_{k+1}^{e} \bigr\Vert _{\Sigma_{f}}\rightarrow0,\qquad \bigl\Vert y_{k}^{e} \bigr\Vert _{\Sigma_{g}}\rightarrow0, \qquad \bigl\Vert Ax_{k+1}^{e}+ By_{k}^{e} \bigr\Vert \rightarrow0, \\ \Vert x_{k+1}-x_{k} \Vert _{S}\rightarrow0, \qquad \Vert y_{k}-y_{k-1} \Vert _{T} \rightarrow0, \\ \Vert x_{k+1}-x_{k} \Vert _{\Sigma_{f}}\rightarrow0, \qquad \bigl\Vert Ax_{k}^{e}-Ax_{k+1}^{e} \bigr\Vert \rightarrow0. \end{gathered} $$

From the boundedness of $\{ \Vert x_{k}^{e} \Vert_{S}\}$, $\{ \Vert x_{k}^{e} \Vert_{\Sigma_{f}}\}$, and $\{ \Vert Ax_{k}^{e} \Vert\}$, we know that $\{ \Vert x_{k} \Vert_{\Sigma_{f}+S+A^{*}A}\}$ is bounded. Furthermore, since $\Sigma_{f}+S+A^{*}A\succ0$ in sPGADM, we get $\{ \Vert x_{k} \Vert\}$ is also bounded. Similarly, we can deduce that $\{ \Vert y_{k} \Vert _{\Sigma_{g}+T+B^{*}B}\}$ is bounded due to the fact that $\{ \Vert y_{k}^{e} \Vert_{T}\}$, $\{ \Vert y_{k}^{e} \Vert_{\Sigma_{g}}\}$, $\{ \Vert Ax_{k}^{e} \Vert\}$, and $\{ \Vert Ax_{k+1}^{e}+ By_{k}^{e} \Vert\}$ are all bounded. Thus, from $\Sigma_{g}+T+B^{*}B\succ0$, $\{ \Vert y_{k} \Vert\}$ is bounded. Also, the boundedness of $\{ \Vert \lambda_{k} \Vert\}$ can come from the boundedness of $\{ \Vert\lambda _{k}^{e}+\sigma(1-\rho)Ax_{k}^{e} \Vert\}$ and $\{ \Vert Ax_{k}^{e} \Vert\}$.

The boundedness of the sequence $\{(x_{k},y_{k};\lambda_{k})\}$ implies that there exists at least one convergent subsequence; for simplicity we denote it as

$$\lim_{k_{i}\rightarrow\infty}\bigl\{ (x_{k_{i}},y_{k_{i}}; \lambda_{k_{i}})\bigr\} = (x_{\infty},y_{\infty}; \lambda_{\infty}),\quad\{k_{i}\}\subseteq\{ 0,1,\ldots\}. $$

By using (17) and (20), we obtain

41 $$\begin{aligned}& A^{*}\lambda_{k_{i}}-S(x_{k_{i}}-x_{k_{i}-1})\in\partial f(x_{k_{i}}), \end{aligned}$$

42 $$\begin{aligned}& B^{*}\lambda_{k_{i}}-\sigma B^{*}(Ax_{k_{i}}+By_{k_{i}}-c)-T(y_{k_{i}}-y_{k_{i}-1}) \in\partial g(y_{k_{i}}). \end{aligned}$$

Because *f* and *g* are closed proper convex functions, the nonempty sets *∂f* and *∂g* are closed. By noticing that $\Vert Ax_{k}+ By_{k}-c \Vert^{2}\leq\Vert Ax_{k+1}^{e}+ By_{k}^{e} \Vert^{2}+ \Vert Ax_{k}^{e}-Ax_{k+1}^{e} \Vert^{2}$, and as mentioned before,

43 $$\begin{aligned}& \bigl\Vert Ax_{k+1}^{e}+ By_{k}^{e} \bigr\Vert \rightarrow0, \qquad \bigl\Vert Ax_{k}^{e}-Ax_{k+1}^{e} \bigr\Vert \rightarrow0, \end{aligned}$$

44 $$\begin{aligned}& \Vert x_{k+1}-x_{k} \Vert _{S}\rightarrow0, \qquad \Vert y_{k}-y_{k-1} \Vert _{T} \rightarrow0, \end{aligned}$$

we take limits with $k_{i}$ on both sides of (41) and (42). It implies that $(x_{\infty},y_{\infty};\lambda_{\infty })$ satisfies the KKT condition:

$$A^{*}\lambda_{\infty}\in\partial f(x_{\infty}),\qquad B^{*} \lambda_{\infty}\in\partial g(y_{\infty}),\qquad Ax_{\infty}+By_{\infty}-c=0. $$

To complete the whole proof, now we will show that $(x_{\infty },y_{\infty};\lambda_{\infty})$ is the unique limit of the sequence $\{(x_{k},y_{k};\lambda_{k})\}$. In fact, since $(x_{\infty},y_{\infty };\lambda_{\infty})$ satisfies the KKT condition, without loss of generality, we can let $(\bar{x},\bar{y};\bar{\lambda})=(x_{\infty },y_{\infty};\lambda_{\infty})$. Thus, from the definition of $\phi _{k}$ in (35), there exists a subsequence $\{ (x_{k_{i}},y_{k_{i}};\lambda_{k_{i}})\}$ such that

$$\lim_{k_{i}\rightarrow\infty}\phi_{k_{i}}=0. $$

Together with the nonincreasing and boundedness of $\{\phi_{k}\}$, we know that $\{\phi_{k}\}$ converges to zero itself. By (35), it turns out that when $k\rightarrow\infty$,

45 $$ \begin{gathered} \bigl\Vert \lambda_{k}^{e}+ \sigma(1-\rho)Ax_{k}^{e} \bigr\Vert \rightarrow0,\qquad \bigl\Vert x_{k}^{e} \bigr\Vert _{S}\rightarrow 0,\qquad \bigl\Vert y_{k-1}^{e} \bigr\Vert _{T} \rightarrow0, \\ \Vert x_{k}-x_{k-1} \Vert _{S}\rightarrow0, \qquad \bigl\Vert Ax_{k}^{e} \bigr\Vert \rightarrow0. \end{gathered} $$

Thus, $\lim_{k\rightarrow\infty}\lambda_{k}=\bar{\lambda}$ since $0\leq\Vert\lambda_{k}^{e} \Vert\leq\Vert\lambda_{k}^{e}+\sigma(1-\rho )Ax_{k}^{e} \Vert+ \sigma|1-\rho| \Vert Ax_{k}^{e} \Vert$. Noticing that $\Vert x_{k}^{e} \Vert_{\Sigma_{f}}\rightarrow0$ in (40), we get

$$\lim_{k\rightarrow\infty}\bigl( \bigl\Vert x_{k}^{e} \bigr\Vert _{\Sigma_{f}} + \bigl\Vert x_{k}^{e} \bigr\Vert _{S}+ \bigl\Vert Ax_{k}^{e} \bigr\Vert \bigr)=0. $$

Thus, from $\Sigma_{f}+S+A^{*}A\succ0$ and $x^{e}_{k}=x_{k}-\bar{x}$, it comes true that

$$\lim_{k\rightarrow\infty}x_{k}=\bar{x}. $$

Also, from (45) together with $\Vert y_{k}^{e} \Vert_{\Sigma _{g}}\rightarrow0$ and $\Vert Ax_{k+1}^{e}+ By_{k}^{e} \Vert\rightarrow0$ in (40), we have

$$\lim_{k\rightarrow\infty}\bigl( \bigl\Vert y_{k}^{e} \bigr\Vert _{\Sigma _{g}}+ \bigl\Vert y_{k}^{e} \bigr\Vert _{T}+ \bigl\Vert By_{k}^{e} \bigr\Vert \bigr)=0. $$

Then, by $\Sigma_{g}+T+B^{*}B\succ0$ and $y^{e}_{k}=y_{k}-\bar{y}$, it deduces that $\lim_{k\rightarrow\infty}y_{k}=\bar{y}$. The above discussion concludes that the whole sequence $\{ (x_{k},y_{k};\lambda_{k})\}$ converges to $(\bar{x},\bar{y};\bar{\lambda })$. The proof is completed. □

## Conclusions

The generalized ADMM, as an important variant of ADMM, is derived from the generalized proximal point algorithm while it is used to solve the sum of maximal monotone operators inclusion problems. Recently, it was shown that the generalized ADMM is also equivalent to the unit step-length ADMM but with additional relaxation steps based on a factor within $(0,2)$. Combining the idea of semi-proximal terms, Xiao et al. [11] proposed a semi-proximal generalized ADMM and numerically illustrated that their proposed method is very promising for semi-positive definite programming. Additionally, Xiao et al. [11] introduced another variant of semi-proximal generalized ADMM with different semi-proximal terms, but its convergence property has not been investigated so far. This study aimed to remedy this deficiency and established its convergence result under some mild conditions in the sense that the relaxation factor is also restricted into $(0,2)$. More precisely, if $\rho\in(0,2)$, theoretical analysis has shown that the proposed algorithm converges globally by assuming that the optimal solutions set is nonempty and the matrices $\Sigma_{f}+{\mathcal {S}}+A^{*}A$ and $\Sigma_{g}+{\mathcal {T}}+B^{*}B$ are both positive definite. The result is quite in accord with the standard semi-proximal ADMM [11]. The paper paid more attention to analyzing the generalized semi-proximal ADMM for solving separable convex minimization. However, it has not been tested with different factor values of *ρ* for performance comparing. This should be our further task to investigate.
